# High prevalence of insulin resistance among Brazilian chronic hepatitis C patients

**DOI:** 10.1590/2359-3997000000315

**Published:** 2017-12-01

**Authors:** Livia Melo Villar, Gabriela Cardoso Caldas, Leticia de Paula Scalioni, Juliana Custódio Miguel, Elisangela Ferreira da Silva, Vanessa Alves Marques, Cristiane Alves Villela-Nogueira, Lia Laura Lewis-Ximenez, Elisabeth Lampe

**Affiliations:** 1 Instituto Oswaldo Cruz Laboratório de Hepatite Viral Rio de Janeiro RJ Brasil Laboratório de Hepatite Viral, Instituto Oswaldo Cruz (Fiocruz), Rio de Janeiro, RJ, Brasil; 2 Universidade Federal do Rio de Janeiro Hospital Universitário Clementino Fraga Filho (HUCFF) Departamento de Clínica Médica Rio de Janeiro RJ Brasil Unidade de Hepatologia, Departamento de Clínica Médica, Hospital Universitário Clementino Fraga Filho (HUCFF), Universidade Federal do Rio de Janeiro (UFRJ), Rio de Janeiro, RJ, Brasil

**Keywords:** Hepatitis C, insulin resistance, prevalence

## Abstract

**Objective:**

This study aims to estimate the prevalence of insulin resistance (IR) among chronic hepatitis C (CHC) patients and their related laboratory and demographic data.

**Subjects and methods:**

In this study, non-diabetic CHC patients referred to Viral Hepatitis Ambulatories from Rio de Janeiro (Brazil) donated blood samples. Insulin was measured using a chemiluminescence immunoassay. IR was determined by HOMA-IR, where HOMA-IR > 2 was defined as IR.

**Results:**

A total of 214 CHC patients were recruited (123 females aged 53.6 years ± 10.9 years). IR was present in 133 patients (62.1%) and was associated in bivariate analysis to higher mean values of age (p = 0.040), triglycerides (p = 0.032), glucose (p = 0.000), insulin (p = 0.000), waist circumference (p = 0.001), and body mass index (p = 0.007); however, none of these variables were significant in the multivariate analysis.

**Conclusions:**

The high prevalence of IR was observed among CHC patients, and there was no difference in clinical or laboratory parameters when both groups were compared in the multivariate analysis. This high IR prevalence could lead to a high risk for development of cardiovascular disease and metabolic disorders.

## INTRODUCTION

Hepatitis C virus (HCV) is a major cause of chronic liver disease, cirrhosis and hepatocellular carcinoma and is responsible for chronically infecting approximately 130 million people worldwide (1). HCV morbidity is not limited to the liver but encompasses extrahepatic conditions, including insulin resistance (IR) and type 2 diabetes mellitus (DM) (2). Chronic infection with HCV is a major risk factor for the development of insulin resistance and, consequently, for type 2 DM (3). High prevalence of type 2 DM was documented among chronic HCV cases compared to the general population or to other chronic liver diseases (2).

Currently, several direct-acting antiviral agents (DAAs) were developed for the treatment of HCV infection (4), but DAAs are not widely available in low resource areas. In these regions, Peg-IFN and ribavirin (RBV) are used for antiviral therapy and result in a sustained virological response (SVR) of approximately 50-80% according to the HCV genotype (5). Low rates of SVR for double therapy are observed in the presence of IR or type 2 diabetes using double therapy (6,7).

Some studies showed that more than 50% of HCV infected individuals have IR (8,9). In Brazil, IR prevalence varies from 20 to 80% in HCV patients (9-13). IR was also associated with age, waist circumference, body mass index (BMI), advanced liver fibrosis (10), HCV viral load (14), higher mean degree of steatosis (15) and higher serum levels of oxidative stress (10,11). Although some studies have reported IR prevalence among HCV individuals, the number of subjects is relatively small, and only one or two HCV genotypes were included. In addition, the relationship among IR and biochemical, haematological, and virological data is unclear. The present study was conducted to determine the prevalence of IR and related demographic and laboratory data among chronic HCV patients referred to hepatology units from Southeast Brazil.

## SUBJECTS AND METHODS

### Study population

A total of 214 consecutive chronic HCV (CHC) patients referred to Viral Hepatitis Ambulatory (FIOCRUZ) and Hepatology Unit (Clementino Fraga Filho University Hospital, UFRJ) were recruited from January 2012 to November 2012 and were included in this study.

The inclusion criteria were individuals of any gender or ethnicity who were more than 18-years-old, presented with reactive serum for anti-HCV/HCV RNA for more than 6 months and agreed to participate in the study after reading and signing the informed consent. The exclusion criteria were the presence of decompensated liver disease (ascites, bleeding from ruptured varices, or encephalopathy), other causes of liver disease (hepatitis B), severe psychiatric disorders, pregnancy or breastfeeding, pancreatitis, the presence of type 2 diabetes mellitus defined as a fasting plasma glucose concentration ≥ 126 mg/dL in two measurements prior to inclusion in the study or previous antiviral treatment. All patients were negative for hepatitis B surface antigen and anti-human immunodeficiency virus.

### Anthropometric and laboratory evaluations

Body mass index (BMI) was calculated as weight divided by the square of the height (kg/m^2^). Waist circumference was measured to the nearest 0.5 cm at the shortest point below the lower rib margin and the iliac crest. Trained staff took these measurements.

Fasting blood samples were obtained for measurement of aminotransferases (alanine aminotransferase, aspartate aminotransferase), alkaline phosphatase, gamma-glutamyltransferase, glucose, total cholesterol, high density lipoprotein cholesterol (HDL cholesterol), very low density lipoproteins (VLDL), low density lipoproteins (LDL) cholesterol, triglycerides, haemoglobin, haematocrit, neutrophil and platelet counts, and thyroid stimulating hormone (TSH).

Serum insulin levels were determined by a chemo-illuminescence immunoassay (Liaison Insulin, Diasorin, Pomezia, Italy) using an autoanalyser. The HOMA-IR score was calculated using the following equation: HOMAIR = fasting insulin x fasting glucose x 0.056/22.5. A HOMA-IR higher than 2.0 was considered insulin resistance (7,15). Fibrosis grade was assessed using aminotransferase (AST) to platelet ratio index (APRI), and an index above 2 indicated the presence of advanced fibrosis (16).

### Virological assays

All patients had positive anti-HCV as measured using EIA (HCV Ab, Radim, Italy) and positive HCV RNA in serum. HCV RNA was detected and quantified using Cobas Taqman HCV (Roche Diagnostics, France), and HCV RNA reactive samples were submitted to the genotype using INNOLIPA HCV II (Innogenetics, Zwijndrecht, Belgium).

### Data analysis

Continuous variables were summarized as the mean ± standard deviation (SD) and categorical variables were described as frequencies and percentages. Comparisons between groups were made by using the Student's *t* test or the Mann-Whitney *U* test for continuous variables and the Chi-square test or Fisher's exact probability test for categorical data. Two-sided P-values 0.05 were considered statistically significant.

Bivariate analysis was done using HOMA-IR as the dependent dichotomous variable (≤ 2 or > 2). Variables significantly associated with IR in the bivariate analysis (p < 0.05) were included in the stepwise multivariate logistic regression model. All data were entered and analysed in SPSS version 20.0.

### Ethical considerations

This study was conducted according to the ethical guidelines of the 2013 Declaration of Helsinki. The protocol and consent form were approved by the Ethics Committee of Fiocruz. All individuals gave their informed consent and answered a questionnaire giving information regarding risk behaviour and sociodemographic data (age, gender).

## RESULTS

### Patients’ characteristics

Table 1 shows the baseline features of the 214 CHC patients included in this study. There were 123 females (57.6%) and their mean age was 53.7 ± 10.9 years. The mean waist circumference was 93.3 cm (±11.8) and the mean BMI was 27.5 kg/m^2^ (± 4.2). The mean HOMA-IR score was 3.4 (3.5) and a HOMA-IR level higher than 2 was found in 133 patients (62.1%). A total of 46 individuals (21.5%) were classified with an advanced grade of fibrosis using the APRI index. HCV genotype was determined among 167 individuals, 145 of them presented with genotype 1 (86.8%) followed by genotypes 3 (9.6%), 2 (2.4%), and 5 (1.2%). After inclusion in the study, 39 individuals underwent therapy with PEG interferon/ribavirin, 16 (41.0%) of whom achieved SVR. The mean values of ALT, AST, and GGT were above the normal values.

**Table 1:** Demographic and laboratory data among Brazilian HCV patients according to insulin resistance (IR) status

Variable	All patients	Without IR N = 81	With IR N = 133	Bivariate analysis P value	Multivariate analysis odds ratio (IC 95%) P value
Age (years)^a^	53.7 ± 10.9	55.64 ± 10.40	52.47 ± 11.16	0.040	0.954 (0.909 – 1.002)
					0.063
Gender (Female/Male)	123/91	47/34	76/57	0.899	NI
Waist circumference (cm)^a^	93.30 ± 11.80	87.96 ± 11.29	95.80 ± 11.24	0.001	1.056 (0.997 – 1.119)
Female gender	92.48 ± 11.26	88.44 ± 10.37	94.23 ± 11.27		0.062
Male gender	94.24 ± 12.49	87.47 ± 12.51	98.00 ± 10.97		
BMI (kg/m^2^)^a^	27.50 ± 4.20	25.90 ± 3.80	28.30 ± 4.20	0.007	1.199 (0.872 – 1.650)
					0.265
Weight categories	57	25	32	0.009	4.572 (0.176 – 118.99)
Healthy weight	108	37	71		0.374
Overweight	49	8	41		
Obese					
Glucose (mg/dL)^b^	90.00 (32.00 – 355.00)	87.00 (32.00 – 24.00)	92.5 (60.00 – 355.00)	0.000	NI
VLDL (mg/dL)^b^	18.00 (7.00 – 115.00)	16.30 (7.00 – 46.80)	19.80 (40.00 – 115.00)	0.059	NI
LDL (mg/dL)^b^	103.00 (14.00 – 1077.00)	97.85 (35.00-1077.00)	107.00 (14.00 – 996.00)	0.154	NI
Triglycerides (mg/dL)^b^	90.00 (34.00 – 573.00)	82.00 (34.00 – 273.00)	99 (40.00 – 573.00)	0.032	1.011 (0.999 – 1.023) 0.071
Total cholesterol (mg/dL)^b^	173.00 (74.00 – 1169.00)	165.50 (100.00-1169.00)	177.00 (74.00 – 1069.00)	0.099	NI
HDL (mg/dL)^b^	49.60 (3.00-130.00)	49.30 (4.00 – 130.00)	49.80 (3.00 – 120.00)	0.844	NI
Insulin (mU/mL)^b^	11.60 (2.00 – 75.00)	6.00 (1.70 – 12.60)	14.70 (6.00 – 75.00)	0.000	NI
ALT (U/L)^b^	52.00 (0.00 – 297.00)	56.00 (12.00 – 269.00)	51.00 (0.00 – 297.00)	0.350	NI
AST (U/L)^b^	62.00 (10.00 – 325.00)	69.50 (21.30 – 244.00)	61.00 (10.40 – 325.00)	0.274	NI
GGT (U/L)^b^	66.15 (10.00 – 1142.00)	73.00 (10.00 – 343.00)	63.00 (14.00-1142.00)	0.399	NI
Alkaline phosphatase^b^	115.00 (6.00 – 95.00)	114.00 (6.00 – 501.00)	117.00 (10.00-695.00)	0.538	NI
TSH (mlU/L)^b^	1.70 (0.07 – 91.40)	1.62 (0.37 – 77.70)	1.75 (0.07 – 91.40)	0.360	NI
Haemoglobina	13.90 ± 1.50	13.84 ± 1.39	13.96 ± 1.53	0.574	NI
Haematocrita	41.60 ± 4.50	41.25 ± 3.57	41.92 ± 4.95	0.297	NI
Platelet (x 10^3^/mm^3^)^a^	188.80 ± 80.10	179.52 ± 78.52	194.54 ± 80.85	0.185	NI
HCV RNA (Ul/mL)^b^	9.80 × 10^4^ (25 – 1.00 × 10^9^)	6.00 × 10^4^ (25 – 3.00 × 10^7^)	2.70 × 10^5^ (28 – 1.00 × 10^9^)	0.108	NI
Fibrosis grade	168 (78.50)	62 (76.54)	106 (79.70)	0.703	NI
Low (APRI < 2), n (%) Advanced (APRI > 2), n (%)	46 (21.50)	19 (23.45)	27 (20.30)		

a Mean ± SD;

b Median, minimum and maximum; NI: not included.

### Baseline HOMA-IR and demographic and laboratory data

When IR was evaluated according to the demographic and laboratory data (Table 1), significant differences were found between the two groups according to age (p = 0.040), waist circumference (p = 0.001), BMI (p = 0.007), weight categories (p = 0.009), glucose (p = 0.000), triglycerides (p = 0.032) and insulin (p = 0.000) in the bivariate analysis. Variables included in the multivariate analysis model were age, waist circumference, BMI, and triglycerides; none of these variables was significantly associated with HOMA-IR. Most individuals with IR had higher mean values of GGT and HCV viral load, but this was not statistically significant.

## DISCUSSION

HCV infection has been related to extra-hepatic manifestations, such as diabetes and IR. The present study demonstrated a high prevalence of IR (62.1%) compared to previous studies conducted in Europe (42-46%) (7,17), North America (40%) (18), Asia (51%) (8) and Brazil (53% and 61%) (9,15). Recently, IR prevalence in the Brazilian Longitudinal Study of Adult Health was found to vary from 12 to 22%, a rate that is lower than that found in the present study (19). In this study, HOMA cut off was 2.0 like used in similar studies that have shown an association of IR and HOMA higher than 2 (20-23). The high prevalence of IR found in the present study could be the result of the large number of HCV individuals who were included. Since the role of insulin resistance and hyperglycaemia as predictors of SVR with DAA HCV treatment has not been clearly established, the high prevalence of IR observed in the present study could have an impact in SVR with DAA treatment. The efficacy of direct-acting antivirals overcame some of the predictors of therapeutic response, but IR and HCV remain important issues to be addressed in the clinical context of the patients.

In the bivariate analysis, IR was associated with age and anthropometric factors (BMI, waist circumference and weight categories); however, this association was not found in the multivariate analysis. Some studies also showed the association of age, waist circumference, and BMI to IR among HCV patients (9,10,11,13,18). IR was also associated with higher mean values of triglycerides and is most likely the result of the characteristics of the HCV replication cycle. The HCV life cycle is associated with cholesterol and lipogenesis pathways in hepatocytes causing enhanced lipogenesis, impaired degradation and impaired export (24).

Some biochemical parameters, such as ALT, AST and, GGT were above the normal values, most likely as a result of chronic liver inflammation. Miyajima and cols. (16) also showed higher mean values of glucose, ALT, AST and GGT among HCV chronic patients compared to non-infected individuals from Japan.

In the present work, HOMA was not associated with HCV viral load, genotype or SVR. Although some studies have shown an improvement in HOMA values among HCV patients presenting with SVR (7) and a high viral load among those presenting with IR (14), other studies have not found any such association (17,25). This lack of association could be the result of the low prevalence of SVR, high viral load and predominance of genotype 1 in the present cohort. One limitation of this study is the low number of non-1 HCV genotypes; other studies that evaluated HOMA- IR in HCV patients also included 75 to 80% of HCV genotype 1 (9,14).

IR was not associated with fibrosis grade as was observed in studies conducted in HCV patients from Northeast and South regions of Brazil (9,14); this could be because of the small number of patients with an advanced grade of fibrosis that was included in these studies.

This study present some limitations: absence of control variables such as, obesity, physical activity and diet; lack of evaluation of patient's diet routine and performance of physical activity and absence of other anthropometric parameters, such as body composition criteria or laboratory evaluation in combination with image, due to financial constraints.

In conclusion, a high prevalence of insulin resistance was observed among HCV patients, demonstrating that this population may be at increased risk for developing type 2 diabetes mellitus. In the bivariate analysis, IR was associated with demographic (age), anthropometric (BMI and waist circumference) and laboratory (glucose, insulin and triglycerides) parameters. Although these variables were not significant in the multivariate analysis, these data suggest a high risk for development of cardiovascular disease and metabolic disorders among HCV patients.
